# ABA flow modelling in *Ricinus communis* exposed to salt stress and variable nutrition

**DOI:** 10.1093/jxb/erw291

**Published:** 2016-07-20

**Authors:** Andreas D. Peuke

**Affiliations:** ADP International Plant Science Consulting, Talstraße 8, D-79194 Gundelfingen, Germany

**Keywords:** ABA, castor bean, flow models, nutrition, phloem transport, signalling, salt stress, stomatal conductance, xylem transport.

## Abstract

In a series of experiments with *Ricinus communis*, abscisic acid (ABA) concentrations in tissues and transport saps, its *de novo* biosynthesis, long-distance transport, and metabolism (degradation) were affected by nutritional conditions, nitrogen (N) source, and nutrient limitation, or salt stress. In the present study these data were statistically re-evaluated, and new correlations presented that underpin the importance of this universal phytohormone. The biggest differences in ABA concentration were observed in xylem sap. N source had the strongest effect; however, nutrient limitation (particularly phosphorus limitation) and salt also had significant effects. ABA was found in greater concentration in phloem sap compared with xylem sap; however, the effect of treatment on ABA concentration in phloem was lower. In the leaves, ABA concentration was most variable compared with the other tissues. This variation was only affected by the N source. In roots, ABA was significantly decreased by nutrient limitation. Of the compartments in which ABA was quantified, xylem sap ABA concentration was most significantly correlated with leaf stomatal conductance and leaf growth. Additionally, ABA concentration in xylem was significantly correlated to that in phloem, indicating a 6-fold concentration increase from xylem to phloem. The ABA flow model showed that biosynthesis of ABA in roots affected the xylem flow of ABA. Moreover, ABA concentration in xylem affected the degradation of the phytohormone in shoots and also its export from shoots via phloem. The role of phloem transport is discussed since it stimulates ABA metabolism in roots.

## Highlight

A meta-analysis of data shows that in *Ricinus* exposed to different nutritional conditions, xylem ABA concentration is better correlated with stomatal conductance, growth, and phloem sap ABA concentration than tissue ABA concentration.

## Introduction

Abscisic acid (ABA) is a major plant signal related to abiotic and biotic stress. The most prominent role of ABA is the effect on stomata due to drought and salt stress (Schachtman and Goodger, 2008; Osakabe *et al.*, 2014). ABA is also paramount for regulation of plant growth and development (Wilkinson and Davies, 2002; Cutler *et al.*, 2010; Antoni *et al.*, 2011). In particular, the impact of ABA on plant growth is well documented for seed development, and root and leaf growth (Wilkinson and Davies, 2002; Hartung *et al.*, 2002; Dodd, 2005; Schachtman and Goodger, 2008; Cutler *et al.*, 2010; Boursiac *et al.*, 2013; Chater *et al.*, 2014).

The definition of a phytohormone—as for all hormones—is a biologically active substance where the site of synthesis is spatially separated from the site of signal perception and effect. Therefore, the transport via the long-distance transport system xylem is pivotal for the effects of ABA on stomata (Sauter *et al.*, 2001; Hartung *et al.*, 2002; Dodd, 2005; Jiang and Hartung 2008; Schachtman and Goodger, 2008; Boursiac *et al.*, 2013; Osakabe *et al.*, 2014). Today the network of ABA transporters and receptors necessary for the transport across membranes and signal perception is well documented [see reviews by Cutler *et al.* (2010) and Boursiac *et al.* (2013)]. The enzymes for *de novo* ABA synthesis are found in the vascular tissues of the vegetative parts of the plants (Antoni *et al.*, 2011; Boursiac *et al.*, 2013). Sauter *et al.* (2001) and Chater *et al.* (2014), for example, showed how conditions in the rhizosphere and atmospheric water conditions modify the transport and compartmentalization of ABA in plant tissue while also highlighting the exchange of ABA in plants with the soil.

*De novo* synthesis of ABA occurs in root tissue, induced by variation in root water potential, soil oxygen content, and degree of soil compaction, and also responding to changes in nutritional conditions in the rhizosphere (Schachtman and Goodger, 2008). Root water potential is an integrating variable that can explain variation in the effects of soil water content on ABA concentration (Puertolas *et al.*, 2013). ABA could also be synthesized in other organs such as the leaves and fruits (Wolf *et al.*, 1990; Wilkinson and Davies, 2002; Dodd, 2005; Jiang and Hartung 2008; Ikegami *et al.*, 2009). Since mineral nutrients are predominantly taken up by the roots, the signal pathway for ABA due to nutrient stress would be the same as the pathway of nutrients to the shoot (Wegner, 2015). Nitrogen (N), phosphorus (P), and potassium (K^+^) are essential nutrients for plant functioning, and their limitation can result in stress reactions (Schachtman and Shin, 2007) that involve ABA.

In the last decades, a series of papers were published that described the effects of nutrition and nutrient limitation as well as salt stress on long-distance transport and partitioning of nutrients and ABA in castor bean (Peuke *et al.*, 1994; Jeschke *et al.*, 1997; Peuke *et al.*, 1998, 2002). In these papers, a specific treatment was compared with a ‘control’. The effect of NH_4_^+^ supply was much stronger on ABA concentrations compared with N limitation, and a moderate salt stress increased ABA transport in NO_3_^−^-but not in NH_4_^+^-fed plants (Peuke *et al.*, 1994). Limitation of P caused low ABA accumulation in leaves and an increased ABA sensitivity of the stomata, despite a massively increased import of ABA in the xylem to the leaves (Jeschke *et al.*, 1997). Similarly, K^+^ limitation led to an increased biosynthesis of ABA in the roots, a nearly 5-fold higher xylem transport, and a doubled phloem transport of ABA (Peuke *et al.*, 2002). In all these papers, the same species was studied under the same growth conditions at the same time, allowing correlations to be evaluated by pooling the data for comparative analysis.

The current study synthesizes and statistically re-evaluates data of ABA concentrations and flows (transport rates in xylem and phloem as well as metabolism of ABA in roots or shoots) throughout whole plants under various growth conditions to provide novel insights. The analyses consolidate changes in ABA concentration in a range of plant tissues (i.e. roots, axes, and leaves) and fluids (i.e. xylem and phloem). As a model species, *Ricinus communis* was used because phloem sap can easily be collected and transport can be modelled. Finally, the inter-relationship and regulatory role of ABA in leaf stomatal conductance, growth, and its own metabolism will be explained and the role of ABA in phloem discussed.

## Materials and methods

### Plant material, cultivation, and drought treatment period

Data used here originate from previous experiments in which *R. communis* plants were cultivated under comparable environmental conditions and experimental design, at the same time (Peuke *et al.*, 1994, 1998, 2002; Jeschke *et al.*, 1997). The principle question in these studies was how ABA concentration and flows within an intact plant are affected by nutritional conditions (up to 11 different treatments) during vegetative growth. Seedlings were transferred to quartz sand culture (one plant in a 5 liter pot) 13 d after sowing and were supplied daily with nutrient solution according to Long Ashton solution (Hewitt, 1966). The water-holding capacity of the quartz sand was ~10% nutrient solution. Different nutritional conditions required some changes in the concentrations of selected compounds; the composition of the nutrient solutions was given in the original papers: (i) different NO_3_^−^ concentrations [0.2 (N limitation), 1.0, 4.0, or 12.0 mM] (Peuke and Jeschke, 1993; Peuke *et al.*, 1994; Jeschke *et al.*, 1996); (ii) NH_4_^+^ (1.0mM) (Peuke and Jeschke, 1993); (iii) low P_i_ or low K^+^ (Jeschke *et al.*, 1996; Peuke *et al.*, 2002); (iv) foliar N (NO_3_^−^ or NH_4_^+^) supply without N in the nutrient solution (Peuke *et al.*, 1998); and (v) with a moderate salt treatment (1.0mM NO_3_^−^ or NH_4_^+^ plus 40mM NaCl) (Peuke and Jeschke, 1995). In these studies the concentrations and flows of carbon (C) and N were modelled.

During growth in the greenhouse, natural light was supplemented by Osram HQL lamps (16h light at 350–500 µmol m^−2^ s^−1^). Temperatures were between 22 °C and 32 °C during the day and between 15 °C and 18 °C during the night. Relative humidity was between 50% and 70%.

### Leaf stomatal conductance

Leaf stomatal conductance of fully elongated non-senescent leaves was measured with a LiCor 1600 steady-state porometer in the glasshouse at a light intensity of 600 mol photons m^−2^ s^−1^, relative air humidity of 55%, and at temperatures between 22 °C and 25 °C. The measurements were carried out between 11:00h and 14:00h.

### Plant harvests

At 41 d after sowing, 7–9 plants of each treatment were harvested. The remaining plants were harvested 10 d later during a second harvest. Plants were divided into roots and shoots. Every plant part was carefully washed with water or sorbitol (50mol m^−3^) in the case of roots to avoid leaching of solutes. Prior to chemical analysis, plant tissue was immediately frozen and lyophilized. The period between the 41st and 51st day after sowing represents the experimental period with exponential growth of the plants. During this time, xylem and phloem saps were collected (see details below) at the site of the hypocotyl from additional plants and at the time of harvest from the harvested plants.

### Plant growth

The lengths of the leaf midribs were measured and, during a period of linear increase within the experimental period, the daily elongation rate (cm d^−1^) was calculated. Within this time, it was usually leaf number 4 that showed this linear development, except under K^+^ limitation where the linear elongation rate was found for leaf 5 and 6.

During the 10 d experimental period between the first and second harvest, the increment of dry weight for leaves, shoots, and roots was calculated based on initial dry weights as:

$$\left({\text{DW}}_{\text{2}.\text{harvest}}-{\text{DW}}_{\text{1}.\text{harvest}}\right)/{\text{DW}}_{\text{1}.\text{harvest}}\left(\text{1}0{\text{ d}}^{-\text{1}}\right)$$

### Sampling of xylem and phloem sap

Phloem sieve tube sap was collected at the time of harvest by shallow incisions into the bark of the hypocotyl according to Pate *et al.* (1974). Xylem sap was obtained as root pressure exudate at the time of harvest and additionally between the two harvesting times (Jeschke and Pate, 1991; Alexou and Peuke, 2013). The shoot was decapitated, ~1cm of bark was removed and washed with deionized water, and a silicon tube was fitted to the stump to avoid contamination with phloem sap. The very first drop of sap was removed and sap was collected for a maximum of 20min. In the case of salt treatment with NH_4_^+^ as the N source, xylem sap collection was not possible with this method. Only the data from root pressure sap are presented as absolute concentration in figures and regression analyses. Additionally, in all treatments, an overpressure (0.1–0.2MPa) above the root water potential (−0.5 to −2.0MPa) was applied to collect enough sap volume for all required analyses, but these data were used only for the model calculations of ([ABA]_xyl_/[N]_xyl_). The transport saps were collected in a greenhouse under ambient conditions between 11:00h and 14:00h.

### ABA analysis

Freeze-dried tissue samples were homogenized and extracted in 80% methanol. Extracts were passed through a Sep Pak C18-cartridge. Methanol was removed under reduced pressure and the aqueous residue partitioned three times against ethyl acetate at pH 3.0. The ethyl acetate of the combined organic fractions was removed under reduced pressure. The residue was taken up in TBS (Tris-buffered saline; 150mol m^−3^ NaCl, 1mol m^−3^ MgCl_2_, and 50mol m^−3^ Tris; pH 7.8) and subjected to an immunological ABA assay (ELISA) as described earlier (Peuke *et al.*, 1994). For phloem and xylem saps, the Sep-Pak C18 purification step was omitted. The aqueous phase after partitioning against ethyl acetate was hydrolysed for 1h at room temperature with 1M NaOH. This fraction was acidified with concentrated hydrochloric acid to pH 3, and partitioned three times against ethyl acetate. The accuracy of the ELISA was verified for *Ricinus* (Peuke *et al.*, 1994). Recoveries of ABA during purification procedures were checked routinely using radioactive ABA and found to be >95%.

### Modelling of flows

Flows of ABA were modelled using previously calculated flows of N in *Ricinus* (see Peuke, 2010), taking into account the assumptions of Jeschke *et al.* (1985). The calculated flows in xylem or phloem (J_ABA,xyl or_ J_ABA,phl_) provide insight into how much ABA is transported independent of water flow rates in the systems.

Assuming mass flow (J) in the xylem and the phloem (Wegner, 2015). the flow of ABA in the xylem (J_ABA,xyl_) is given by the flow of nitrogen (J_N,xyl_) and the molar ratio [(ABA/N)_xyl_]:

$${\text{J}}_{\text{ABA},\text{xyl}}={\left(\text{ABA}/\text{N}\right)}_{\text{xyl}}\times {\text{J}}_{\text{N},\text{xyl}}$$

Similarly, the phloem flow (J_ABA,phl_) is:

$${\text{J}}_{\text{ABA},\text{phl}}={\left(\text{ABA}/\text{N}\right)}_{\text{phl}}\times {\text{ J}}_{\text{N},\text{phl}}$$

The differences between net flows of ABA toward or from an organ and its increment (Δ_ABA_) in that organ yield the net metabolic removal (degradation) or formation (synthesis) of ABA:

$${\text{J}}_{\text{ABA},\text{met}}=\Delta_{\text{ABA}}+{\text{J}}_{\text{ABA},\text{xyl}}+{\text{J}}_{\text{ABA},\text{phl}}$$

If net metabolic change (J_ABA,met_) is negative, net degradation is taking place; if it is positive, net synthesis occurs. However, these estimates of net synthesis or degradation are the balance over the experimental period of 10 d, which may include continuing synthesis and degradation in the same organ. For the root, it will also embrace the leaching of ABA to the rhizosphere.

To demonstrate principal mechanism and for presentation of ‘general’ flow models depending on different nutritional conditions, the flow data of the summarized studies (Peuke *et al.*, 1994, 1998, 2002; Jeschke *et al.*, 1997) were re-evaluated by regression analysis (see below). The effect of metabolism [(bio-)synthesis or degradation] on xylem and phloem flows as well as increments in root and shoot were tested (additionally the effect of the xylem flow on increment in shoots and phloem flow). The generalized flows per plant are given as 100% of metabolism ±SE of the estimated slope of the regression in the models.

### Statistics

ABA concentration in the above- and below-ground plant fractions as well as leaf stomatal conductance were obtained from 7–9 plants, cultivated in individual pots in each single treatment. Concentrations of ABA in xylem (collected under root pressure) and phloem saps and in tissues are given as means ±SE.

Statistical calculations were performed with SAS (V9.2 and V9.4, SAS Institute Inc., Cary, NC, USA), and non-linear regression analysis with Sigma Plot (V12.5, Systat Software Inc., San Jose, CA, USA).

One-way ANOVAs (model: ‘treatment’) were performed using the general linear model (GLM) procedure. The adjustment of multiple comparisons according to the Tukey test was chosen for the *P*-values and confidence limits for the differences of least squares of the means (LS-means). Additionally a three-way ANOVA (model: ‘N source’ ‘limitation’ ‘salt’) was performed. For this purpose, the data set was divided by N source (NO_3_^−^ versus NH_4_^+^), limitation of nutrients (0.2mM NO_3_^−^, foliar application, P and K^+^ limitation versus 1, 4, and 12mM NO_3_^−^), and application of salt.

Regression analyses (REG procedure of SAS) were used to test a linear model which included an intercept (*y*=*a*+*b*×*x*). If the intercept was not significant (H_0_: estimates=0), but, the slope was and additionally *r* ^2^ >0.5, a model without intercept was calculated (*y*=*b*×*x*). In models with no intercept, *r* ^2^ was redefined. In the figures only significant estimates (slope as well as intercept) are presented; non-significant estimates or models were eliminated and minimum *r* ^2^ was set at 0.5. Mean values of the different treatments or studies were used for regression analyses. Generally, it was tested if the concentration in the tissue or in the transport sap, or the transport rate (=flow or delivery) of ABA fit better as independent variables (or ‘predictors’) to the dependent variable. For example: is the stomatal leaf conductance better correlated to leaf ABA concentration, xylem sap ABA concentration, or xylem ABA flow (transport to the leaves; see Fig. 3)?

## Results

### ABA in xylem and phloem sap and plant tissues

**Fig. 1.**
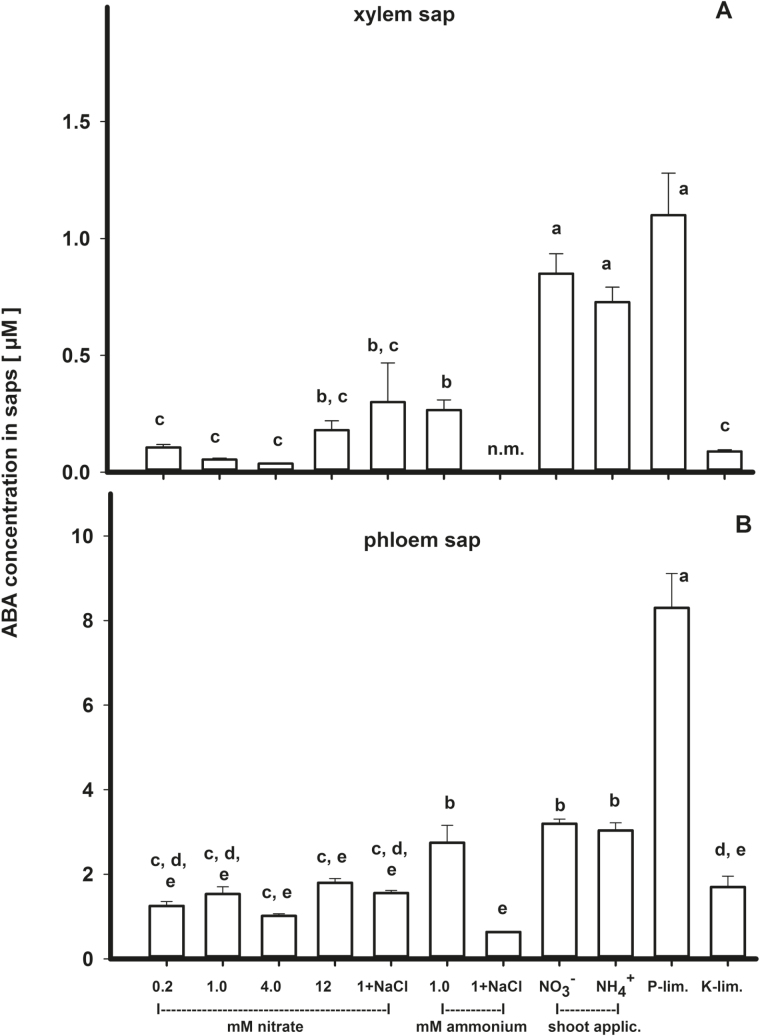
Concentration of ABA in xylem (A, *n*=5–28; collected by root pressure) and phloem sap (B, *n*=4–32; collected from the hypocotyl) of *Ricinus communis* grown under different nutritional conditions (see the Materials and methods for details) 41–51 d after sowing. Given are the means and SEs; homogenous groups are indicated by lower case characters. Nitrate nutrition, 0.2, 1.0, 4.0, or 12.0mM NO_3_^−^; 1+NaCl, 1.0mM NO_3_^−^+40mM NaCl; ammonium nutrition, 1.0mM NH_4_^+^; 1+NaCl, 1.0mM NH_4_^+^+40mM NaCl; NO_3_^−^/NH_4_^+^, shoot application by spraying with NO_3_^−^ or NH_4_^+^ solution; P-lim., phosphorus limitation; K-lim., potassium limitation; n.m., not measured.

The highest ABA concentration in xylem sap was observed in *Ricinus* after the nutritional treatment with both foliar application of N (NO_3_^−^ or NH_4_^+^) and P limitation (Fig. 1A). The lowest ABA concentration in xylem sap was observed after NO_3_^−^ was supplied as the N source. Ammonium as the N source, nutrient limitation, and salt supply significantly increased xylem ABA concentration (Table 1). In general, in xylem sap of *Ricinus* supplied with NH_4_^+^, ABA was much higher compared with sap of plants that were supplied with NO_3_^−^ (0.21±0.03 versus 0.11±0.02 µM). Similarly, nutrient limitation generally enhanced the concentration of ABA in xylem sap (0.21±0.03 µM) when compared with sufficient supply with nutrients (0.11±0.02 µM). However, the effect of increasing the ABA concentration following nutrient deficiencies was less pronounced after N and K^+^ limitation. Due to the method applied for collecting xylem sap—root pressure—the concentration may be overestimated compared with those in a transpiration stream with higher flow rates. On the other hand, this method used a ‘natural’ reaction of the plant compared with artificial applied pressure. Within the maximum sap collection time of 20min, the absence of supply via phloem due to decapitation will not play a role. On average, the concentrations of ABA in root pressure saps were higher (0.181±0.012 µM) but correlated very well with those collected following pressurization (0.049±0.004 µM, Pearson correlation coefficient *K*_P_=0.981; data not shown).

**Table 1:** Summary of one way ANOVA (model: ‘treatment’) and three-way ANOVA (model: ‘N source’ ‘limitation’ ‘salt’) of ABA concentrations

	One-way ANOVA	Three-way ANOVA					
	Treatment	N source		Limitation		Salt	
		NO_3_^−^	NH_4_^+^	Yes	No	Yes	No
Xylem	***	***		***		NS	
(µM)		0.11±0.02	0.21±0.03	0.21±0.03	0.11±0.02	0.19±0.04	0.13±0.01
Phloem	***	***		***		NS	
(µM)		1.74±0.10	2.81±0.19	2.67±0.15	1.88±0.13	2.40±0.20	2.16±0.10
Leaves	***	**		NS		NS	
(nmol g^−1^ DW)		2.38±0.39	4.29±0.54	3.77±0.54	2.90±0.40	3.08±0.71	3.58±0.30
Axis	***	NS		NS		*	
(nmol g^−1^ DW)		2.66±0.48	3.77±0.62	3.71±0.65	2.71±0.47	4.17±0.82	2.27±0.38
Roots	***	NS		**		NS	
(nmol g^−1^ DW)		0.85±0.16	0.55±0.20	0.31±0.21	1.09±0.15	0.49±0.26	0.91±0.12

Given are the LS-means ±SE; significant differences are indicated by asterisk: *P<0.05; **P<0.01; ***P<0.001 (NS, not significant).

The concentration of ABA in phloem sap was >10-fold higher compared with that detected in xylem sap (1.8±0.97 versus 0.14±0.21 µM; Fig 1B). The highest concentration of ABA in the phloem was detected in plants that were exposed to P limitation (Fig. 1B). All other treatments resulted in similar concentrations of ABA in phloem. Similar to the effects of salt application on ABA concentration in xylem, these treatments together with limited supply of nutrients also resulted in increased ABA concentration in the phloem (Table 1).

**Fig. 2.**
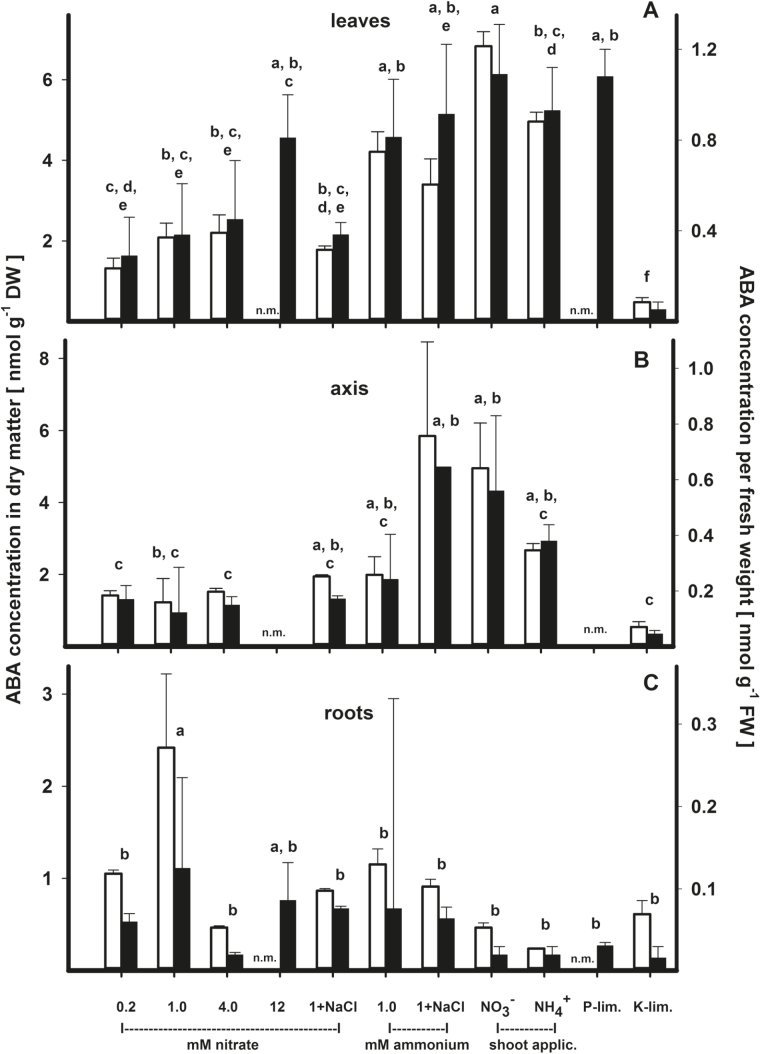
Concentration of ABA per dry (open columns) and fresh weight (filled columns) in (A) leaves, (B) axis, and (C) roots of *Ricinus communis* grown under different nutritional conditions 41 d after sowing. Given are means and SEs (*n*=7–9). For further details, see the legend of Fig. 1; homogenous groups were calculated for dry matter data.

The concentration of ABA relative to tissue dry weight followed a clear pattern, where leaves (3.13±2.21 nmol g^−1^ DW) > axis (2.42±2.62 nmol g^−1^ DW) > roots (0.88±0.90 nmol g^−1^ DW; Fig. 2). In leaves, a number of significant statistical differences were observed, of which the most distinct observation was the low concentration of ABA after K^+^ limitation (Fig. 2A). However, on the whole, limitation of ther nutrients and salt supply did not increase ABA in leaves (Table 1). The supply of NH_4_^+^ significantly increased the ABA concentration in leaves (4.29±0.54 nmol g^−1^ DW), compared with the supply of NO_3_^−^ (2.38±0.39 nmol g^−1^ DW). While nutrient treatments produced significant differences in the concentration of ABA in axis tissue, supplying the plants with different sources of N did not result in similar effects (Fig. 2B). The concentration of ABA in root tissues was generally low, and no clear trends in these concentrations were discernible regardless OG whether expressed per unit fresh or dry weight of plant material (Fig. 2C).

### Correlations of ABA with leaf stomatal conductance and plant growth

**Fig. 3.**
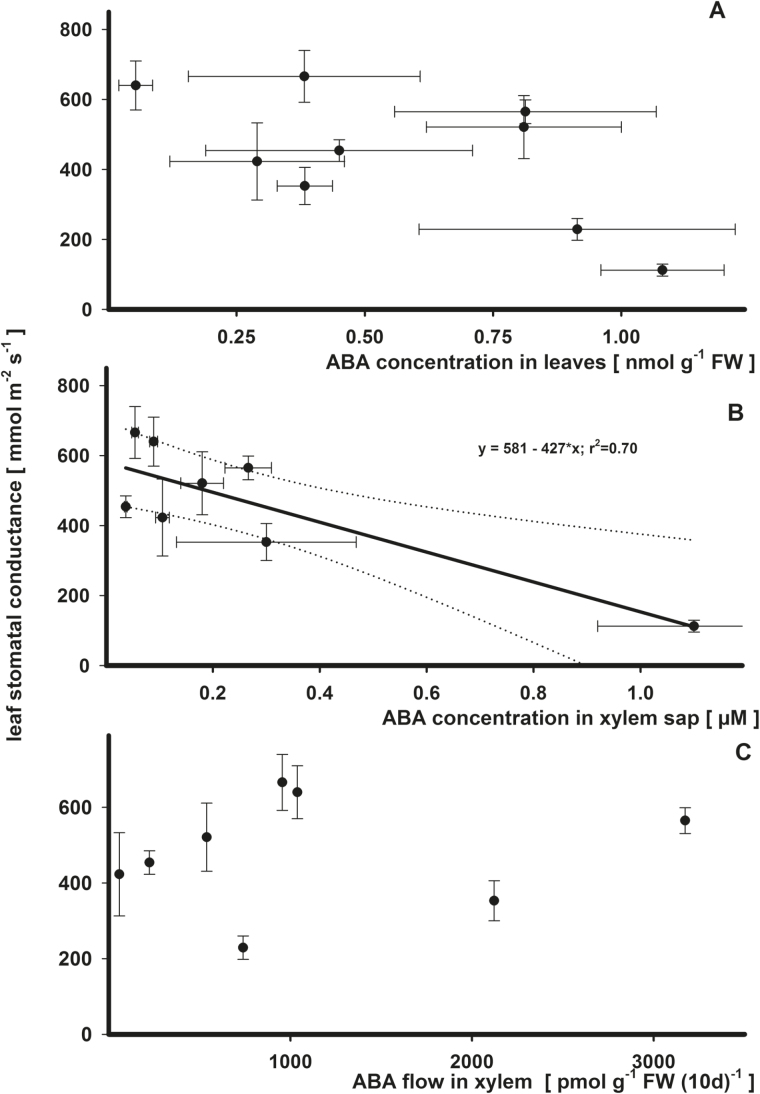
Regression analysis for the effects on leaf stomatal conductance by ABA in leaves (A), by ABA in xylem sap (B, root pressure), and by ABA flow in the xylem from roots to leaves (C) in *Ricinus communis* in a vegetative growth period 41–51 d after sowing. For the calculations, the means of the summarized studies/treatments were used. For further details, see the legend of Fig. 1. Significant relationships (H_0_, estimate=0; *P*<0.05) are indicated by the solid bold line (*r* ^2^>0.50).

Xylem ABA concentration was better correlated (*r* ^2^=0.70; Fig. 3B; Supplementary Table S1 available at *JXB* online) with stomatal conductance than foliar ABA concentration (*r* ^2^=0.31; Fig. 3A). With theoretical zero ABA in xylem sap, the leaf stomatal conductance would be 581 mmol m^−2^ s^−1^ (intercept) and per 0.1 µM ABA in the xylem sap the leaf stomatal conductance would decline by 42.7 mmol m^−2^ s^−1^.

**Fig. 4.**
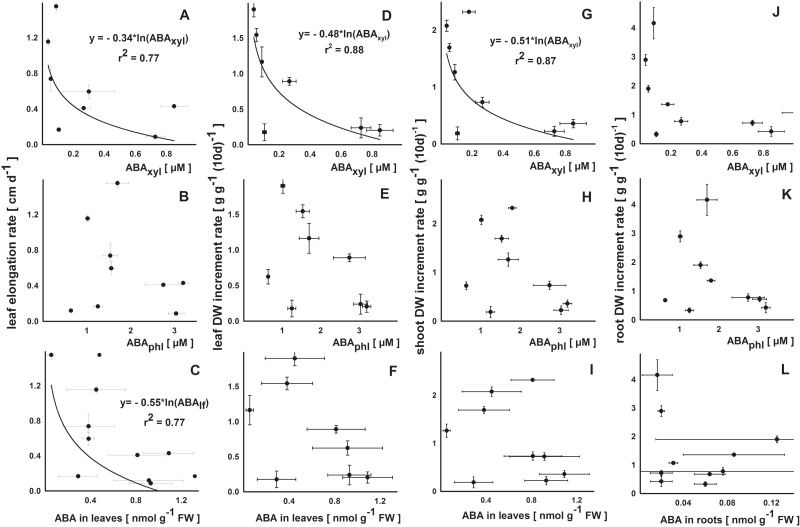
Regression analysis for the effects of ABA in xylem sap (root pressure: A, D, G, and J), in phloem sap (B, E, H, and K), and in leaf or root dry matter (C, F, I, and L) on leaf elongation rate, DW increment rate (A–C and D–F), shoot DW increment rate (G–I), and root DW increment rate (J–L) in *Ricinus communis* in a vegetative growth period 41–51 d after sowing. For the calculations, the means of the summarized studies/treatments were used.

No linear relationship was found for ABA concentration in any type of tissue or sap collected to that of the calculated growth parameters that included leaf elongation rate, and leaf, shoot, and root dry matter increment (Fig. 4; Supplementary Table S2). However, some curvilinear relationships were observed where the leaf elongation rate was negatively correlated with ABA concentration in xylem sap (*r* ^2^=0.77; Fig. 4A) and in leaf material (*r* ^2^=0.77; Fig. 4C). Similar relationships were detected for the effect of ABA concentration in xylem sap on growth rates of leaves (*r* ^2^=0.88; Fig. 4D) and shoots (*r* ^2^=0.87; Fig. 4G). No statistically significant effects of ABA concentrations or flows on root increment were observed (Fig. 4J–L; Supplementary Table S2).

### Potential dependence of phloem ABA on leaf and xylem ABA

**Fig. 5.**
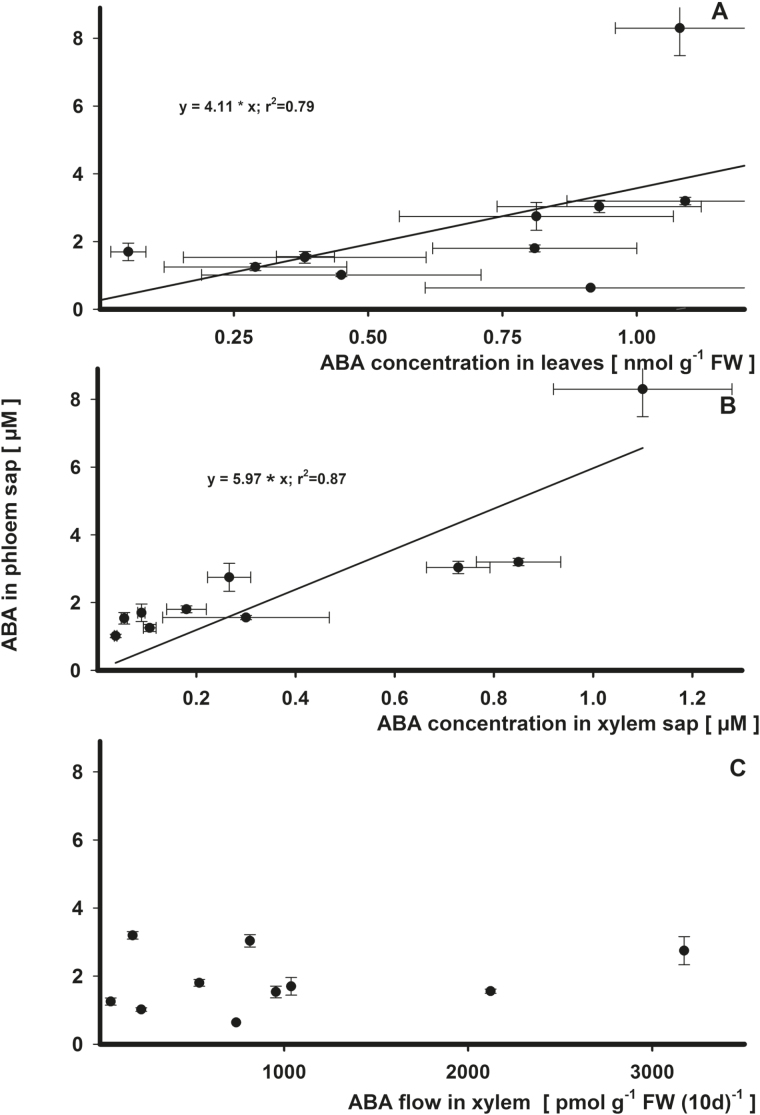
Regression analysis for the effects on ABA concentration in phloem sap by ABA in leaves (A), by ABA in xylem sap (B, root pressure), and by ABA flow in the xylem from roots to leaves (C) in *Ricinus communis* in a vegetative growth period 41–51 d after sowing. For the calculations, the means of the summarized studies/treatments were used. Significant relationships (H_0_, estimate=0; *P*<0.05) are indicated by a solid bold line (*r* ^2^>0.75).

ABA in phloem sap varied in proportion TO the concentration in leaves (*r* ^2^=0.79; Fig. 5A; Supplementary Table S1B). In comparison, ABA concentration in xylem correlated with that measured in phloem (*r* ^2^=0.87; Fig. 5B). A slope of 5.97 indicates that an increase in xylem-transported ABA will lead to a 6-fold accumulation of ABA in phloem. No correlation existed between the flow of ABA in xylem and phloem concentration (Fig. 5C).

### Potential ABA effects on its metabolism in roots

**Fig. 6.**
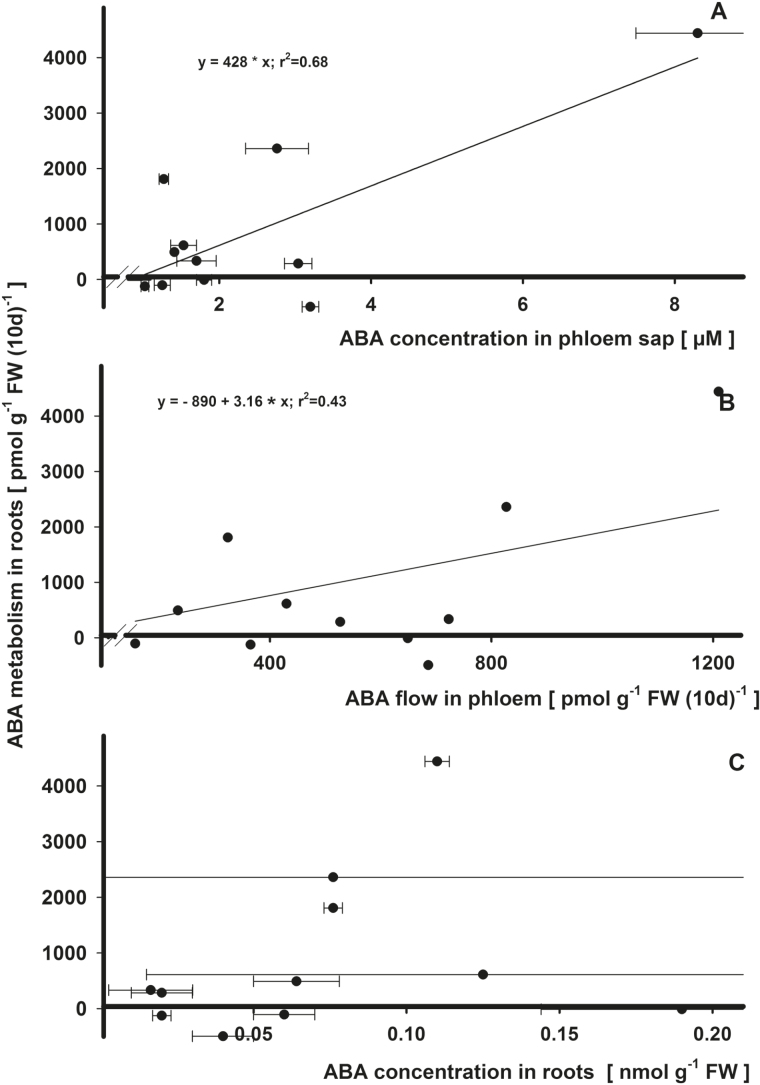
Regression analysis for the effects on ABA metabolism (synthesis or degradation) in roots by ABA in phloem sap (A), by ABA flow in phloem (B), and by ABA in roots (C) in *Ricinus communis* in a vegetative growth period 41–51 d after sowing. For the calculations, the means of the summarized studies/treatments were used. Significant relationships (H_0_, estimate=0; *P*<0.05) are indicated by a solid bold line (*r* ^2^>0.5).

ABA concentration in phloem sap at the hypocotyl was positively and significantly (*r* ^2^=0.68, Supplementary Table S1C) proportional to ABA metabolism in the roots (Fig. 6A). In addition, this analysis provides evidence that ABA degradation occurred in roots. On average, *de novo* synthesis of ABA in roots was positive when the ABA concentration in phloem was >0.85 µM. The ABA flow in the phloem to the roots pointed to a similar positive effect on ABA *de novo* synthesis in roots (*r* ^2^=0.53, Fig. 6B). There was no effect of root ABA on its metabolism (Fig. 6C).

### General flow model for ABA

Regardless of plant treatments, the average flow rates of ABA over the course of the experiment (days 41–51) show a net synthesis of ABA in roots (872±440 pmol g^−1^ FW 10 d^−1^) and a degradation of ABA in the shoots (−662±410 pmol g^−1^ FW 10 d^−1^) when based on a fresh weight basis of the whole plant (Fig. 6A). Rates of ABA transported in xylem (1444±537 pmol g^−1^ FW 10 d^−1^) exceeded those of *de novo* synthesis in roots, in part due to recycling of ABA from the phloem (557±92 pmol g^−1^ FW 10 d^−1^) back into root tissue. In addition a low increment of ABA in shoots (107±25 pmol g^−1^ FW 10 d^−1^) and roots (16±7 pmol g^−1^ FW 10 d^−1^) was found. However, this increment was low compared with the metabolism and flow of ABA throughout the plant. Due to the different treatments, the variations of values was very high.

A clear correlation between synthesis of ABA in the roots and export via xylem was found (Fig. 6B). All ABA originating from *de novo* synthesis and reloading processes (ABA from root cells or recycled via phloem) in roots (105% of *de novo* synthesis plus 441 pmol g^−1^ FW 10 d^−1^) was transported to the shoot. In the shoot, 73% of the ABA import via xylem (plus 392 pmol g^−1^ FW 10 d^−1^) was degraded. The xylem flow additionally affected the phloem flow significantly (13% of xylem flow plus 371 pmol g^−1^ FW 10 d^−1^). On the other hand, the root increment of ABA was affected by the phloem flow (7% minus 24 pmol g^−1^ FW 10 d^−1^). No correlations of shoot metabolism with other flows were detected.

## Discussion

### ABA concentration in tissues and saps

ABA is well known as a signal in relation to drought and salt in plants (Schachtman and Goodger, 2008; Osakabe *et al.*, 2014). The present study shows that different treatments used to impose nutrient deficiencies on *Ricinus* produced similar effects on ABA concentrations throughout a range of plant tissues. For example, ABA was increased in leaves and axis tissue due to NH_4_^+^ supply and similarly in axis tissue by N foliar application. Similarly to the values reported here, Zdunek and Lips (2001) and Vysotskaya *et al.* (2008) found an increase of ABA in leaves rather than in roots due to general nutrient limitation in pea and durum wheat. Yet other studies showed that the concentration of ABA increased more uniformly throughout plant tissues either of vegetative maize grown under depleted availability of N, P, K, and sulfur (S) (Battal *et al.*, 2003) or in vegetative pea exposed to lime-induced P deprivation (Rothwell *et al.*, 2015).

It is further shown that variation in ABA concentration as a consequence of these treatments was more pronounced in saps than in tissues. ABA concentration in phloem varied 13-fold (0.64 to 8.30 µM, in NH_4_^+^ treatment compared with P limitation), whereas in xylem this variation was even more pronounced and varied by a factor of 29 (0.04 to 1.10 µM, NO_3_^−^ treatment compared with P limitation). In roots and axes, this factor was only ~10, yet in leaves it was somewhat closer to the range reported for phloem sap. There was a positive correlation between xylem and leaf ABA (*y*=0.34+0.78*x*, *r* ^2^=0.67, data not shown).

While the variation of ABA throughout all treatments was most pronounced in xylem sap, the total concentration of ABA in phloem sap was ~10-fold higher compared with that in xylem sap. This observation points to a trapping of ABA in phloem sap. The strongest effect on ABA in xylem sap was caused by NH_4_^+^ compared with NO_3_^−^ nutrition (4-fold; Table 1). Limitation of other nutrients or application of salts only triggered a 2-fold variation of ABA concentration in tissues and saps. More generally, the present study illustrates that the salt effect was strongly influenced by the N source, since *Ricinus* is moderately salt tolerant but sensitive to NH_4_^+^ nutrition (Jeschke and Pate, 1991; Peuke and Jeschke, 1993). Additionally, a strong effect of nutrient limitation on ABA was observed in roots (reduction to 25% of the non-limited value), with a smaller effect observed due to salt in the axis material (2-fold increase).

The type of N source provided—NH_4_^+^ versus NO_3_^−^—has very different effects on plant metabolism and associated regulation of ABA. The N source determines ionic balances in root tissues that in turn affect the intracellular pH of the roots and rhizosphere. Furthermore, the N source will trigger a range of different metabolic responses and impacts the energetic efficiency of N uptake that is generally more costly when assimilating NO_3_^−^ (Pfautsch *et al.*, 2009; Wegner, 2015). Ammonium is readily assimilated into organic compounds in roots, while with increasing supply of NO_3_^−^ greater amounts of this inorganic form of N are transported to leaves where they will be reduced. These processes affect apoplastic and rhizosphere pH. In this regard, the influence of long-distance transport on ABA balance and gradients is of special interest. Schachtman and Goodger (2008) pointed out that pH in different compartments plays a central role in determining the physiological effects of ABA. Wilkinson and Davies (2002) demonstrated that xylem pH affected compartmentation of ABA, with greater apoplastic concentrations when the sap was alkaline. It was speculated that increased N supply led to a decrease in stomatal conductance in maize, mediated by pH effects on ABA distribution (Wilkinson *et al.*, 2007). The pH in the apoplast of nitrate-fed plants is higher compared with that of ammonium-fed plants; alkalization increased further with increasing nitrate supply, and thus the redistribution of ABA is reduced and the intensity of the ABA signal remains high (Jiang and Hartung, 2008).

### Leaf stomatal conductance and growth: leaf or xylem sap ABA?

Increased ABA concentrations have been correlated with decreased stomatal conductance, and the relative importance of ABA in different plant tissues in eliciting stomatal closure has been discussed (Wilkinson and Davies, 2002). It was shown here that the ABA concentration in the xylem sap had the strongest correlation with leaf stomatal conductance when compared against other potential sources such as ABA in leaf tissue or xylem flow of ABA. On the one hand, the higher strength of the correlation in Fig. 3B is driven by an influential point with xylem ABA concentration >1 µM; however, the slopes for leaf ABA or ABA xylem flow were never significant (Supplementary Table S1A). Similar correlations between xylem sap ABA concentration and leaf conductance have been described elsewhere (Gowing *et al.*, 1993; Trejo *et al.*, 1995; Wilkinson and Davies, 2002; Dodd, 2005; Jiang and Hartung, 2008). While increasing concentrations of ABA in leaf tissue had a similar tendency to reduce stomatal conductance, they were not statistically significant. Due to the effect of ABA on leaf stomatal conductance and as a consequence of reduced transpiration, ABA may affect its own transport via lower water xylem flow (Schachtman and Goodger, 2008). On the other hand, xylem ABA concentration increased due to lower transpiration while enhanced flow of ABA in the xylem indicates a supply of root-sourced ABA to the leaves. For this problem the flow of ABA in xylem suggests the supply of root-borne ABA to the leaves. However, the flow of ABA in the xylem to the leaves was not correlated to leaf stomatal conductance (Fig. 3C; Supplementary Table S1A). In contrast, some reports also indicate that the amount/flux of ABA delivered to leaves or stomata does have a significant effect on stomatal conductance: in *Prunus* (Gowing *et al.*, 1993) or *Commelina* and *Phaseolus* (Trejo *et al.*, 1995). ABA flows in the present study integrate a 10 d experimental period. In contrast, the response of stomata to abiotic stresses is very sensitive and occurs within very short time scales, which may be the reason for the lack of correlation between stomatal conductance and ABA flow. Notwithstanding xylem ABA concentration, that its concentration in leaf tissue had a similar but statistically not significant effect points to a well-balanced import and degradation of ABA in leaves.

**Fig. 7.**
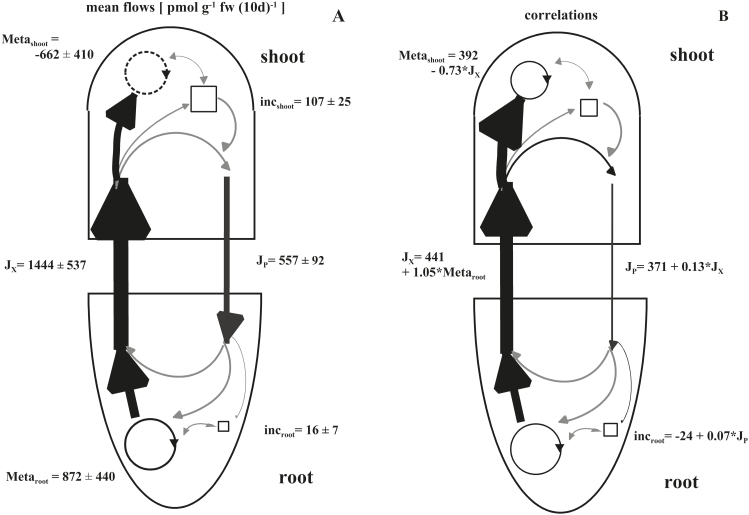
Flow profiles for transport, metabolism, and accumulation of ABA in shoot and root of *Ricinus communis* in the vegetative growth period 41–51 d after sowing. (A) Average values ±SE [pmol g^−1^ fw (10 d)^−1^], and (B) correlations/general flow models following a regression analysis of flow values from models on 11 different nutritional conditions. The arrows on the left side indicate flow in the xylem (J_X_), and on right side flow in the phloem (J_P_). The squares indicate the increment in shoot or root (inc_shoot/root_), and the circle, the metabolism: (+) *de novo* synthesis, (−) degradation (Meta _shoot/root_). Black arrows indicate verifiable (provable) flow values or relationships which were statistically significant (*r* ^2^>0.5, *P*<0.05); grey arrows indicate potential exchange.

The mean flows of ABA within *Ricinus* over a 10 d period show a relatively high degree of variation in all parameters. Averaged over all treatments, the root was clearly the site of ABA net synthesis (872±440 pmol g^−1^ FW 10 d^−1^) and the shoot the place of degradation (−662±410 pmol g^−1^ FW 10 d^−1^); accumulation was very low in both organs compared with transport rates (see Fig. 7A). Root ABA synthesis was correlated with ABA flow, with almost all root-synthesized ABA exported to the shoot. Secondly, ~73% of xylem-imported ABA was degraded in shoots, and ~13% of xylem-borne ABA is recycled via phloem to the roots. Other significant correlations between flows, metabolism, or increments were not found (e.g. between metabolism in the shoot and phloem flow or shoot increment). About 400 pmol ABA g^−1^ FW 10 d^−1^ was present in flows and shoot metabolism. Jiang *et al.* (2012) studied ABA flows in pea and found that in control plants 60% of imported ABA was degraded in shoots and 38% recycled in the phloem back to the roots. However, due to an inoculation with the rhizobacterium *Variovorax paradoxus*, these relationships were changed to 32% (degradation) or 67% (recycling), respectively. In *Lupinus*, ABA net synthesis apparently occurred under control conditions, since 104% of ABA import via the xylem was recycled back; however, salt treatment increased xylem ABA flow and degradation in the shoot while only 44% was recycled (Wolf *et al.*, 1990)

Due to a lower availability of water, plants can reduce water loss by closing stomata and slowing down leaf expansion (Wilkinson and Davies, 2002). An increased leaf xylem ABA concentration was significantly correlated with decreased leaf dry matter increment, leaf elongation, and shoot increment (Fig. 4A, D, G). The sole significant effect of leaf ABA on growth was found for the leaf elongation rate (Fig. 4C). Phloem ABA concentrations were not correlated with leaf growth parameters or root dry matter increments.

### ABA transport in the phloem

Previously, a tight correlation between ion concentration in leaf tissue and phloem sap was observed (Peuke, 2010). However, this correlation was not as pronounced for ABA (*r* ^2^=0.79 calculated without intercept, Fig. 5A; Supplementary Table S1B). In contrast, a highly significant effect of ABA in xylem sap on ABA concentration in phloem sap was detected in *Ricinus* (*r* ^2^=0.87, Fig. 5B). Vysotskaya *et al.* (2008) also found increased ABA concentrations in phloem exudates due to nutrient limitation and explained the observed higher ABA concentration in root tips of wheat by the import via the phloem.

The present study provides evidence for significant amounts of ABA in the phloem sap. As result of mass flow, it is most likely that ABA in the phloem will be transported to the sinks. The inference of this observation raises the question of the role of ABA transport in the phloem. (i) Can ABA in phloem be translocated to growing shoots? (ii) Does ABA play a different regulatory role in phloem compared with xylem? (iii) Does ABA phloem transport contribute to root–shoot signalling by removing ABA from the shoot? (iv) Why would ABA recirculated to the root?

Regarding question (i), Sauter *et al.* (2001) concluded that recirculation of ABA in the phloem may be an important signal to the shoot. It may indeed also be possible that mature leaves send signals to developing leaves (Chater *et al.*, 2014). In principle, a molecule can travel by diffusion in the apoplastic or symplastic space of plant tissue albeit slowly. In the dimension of some cells, this might be sufficient (calculated for hexose, van Bel and Hess, 2003). Kramer (2006) estimated that the decay length (the distance over which the hormone concentration decreases by a factor of 10) is several magnitudes lower in the apoplastic space (35–110 µm) compared with the flowing xylem sap, depending on the apoplastic dimension or xylem flow speed, respectively. Therefore, an ABA molecule can travel only a limited distance within the apoplastic space, if not carried in the xylem by the transpiration stream. Bruggemann *et al.* (2001) modelled the diffusive transport of phytohormones through the aleurone layer of germinating barley grain and found that ABA—in contrast to gibberellin—was not transported and remained at the site of synthesis. Based on these considerations above, it is very unlikely that ABA can diffuse to growing parts of plants, but must be transported in the phloem. Therefore answering the question ‘Can ABA in phloem be translocated to growing shoots?’ can be achieved, simply by trapping from xylem into phloem [where both systems are not very well isolated, see also question (ii) below].

In order to address the second question, a more general question arises as to whether an ABA molecule can move outside a transport system, thereby playing different regulatory roles in the xylem and phloem. First, due to the tight spatial location of phloem and xylem and the pH gradient between both compartments (alkaline compared with acidic, respectively), weak acids will be trapped in the phloem (Kramer, 2006; Boursiac *et al.*, 2013). Therefore, the existence of ABA in the phloem sap may be simply due to a trapping effect without physiological relevance. Due to the observed tight correlation between xylem and phloem ABA (Fig. 5B), the signal from xylem can easily be ‘translated’ to the phloem and thereby reach growing plant parts less connected to the xylem pathway. However, in the present study, the statistical analysis of ABA concentration in phloem sap with either leaf or root growth did not produce meaningful correlations (Fig. 4B, E, H, K). However, the composition of the phloem sap at the hypocotyl (as collected and measured here) must not be similar to that in shoot tips, particularly with respect to ABA concentrations. Jeschke *et al.* (1997) showed that in *Ricinus* the ABA:C ratio in phloem sap from petioles decreased from 1.1 to 0.3 (nmol mmol^−1^) with increasing leaf age. Therefore, a statistical analysis with ABA concentration from phloem saps of the shoot tip with shoot growth may result in better correlations. Thus, it is not very likely that ABA plays a different regulative role in phloem compared with xylem within the shoot.

Regarding the third question, phloem transport of ABA from shoot tissues has the potential to make a significant contribution to the regulation of growth and physiology. The mean flow model revealed that 13% of the xylem ABA is recycled back to the roots via phloem (Fig. 7B), but, this is only a fraction compared with 73% of xylem ABA degraded in shoots. Therefore, the contribution of ABA recycling via phloem for removing ABA from the shoot is relatively low. This result suggests that leaves reduce ABA concentrations via metabolism (degradation) and that the capacity for this process is sufficient to provide enough regulatory control over appropriate time frames to sustain growth and development.

Finally, to the last question: Why would ABA be recycled to root tissues? Would this process be energetically efficient in replacing ABA synthesis? In contrast to the effect on root growth (see above), a correlation between ABA phloem concentration and metabolism was observed (Fig. 6A). At the site of phloem sap collection—the hypocotyl—there is a clear basipetal transport direction to the root, with phloem and xylem clearly separated by the cambium. This observation could therefore be interpreted as a positive feedback. Cornish and Zeevaart (1985) demonstrated root ABA accumulation in *Xanthium* and tomato prior to leaf wilting. If transport of ABA to the roots via phloem was inhibited by stem girdling, accumulation of ABA in roots of beans was prevented in response to chilling (Vernieri *et al.*, 2001). Ikegami *et al.* (2009) showed that in Arabidopsis drought-stressed leaves were a source of ABA, and transport to the roots was activated. Manzi *et al.* (2015) demonstrated in tomato and citrus by stem-girdling and grafting experiments that accumulation of ABA in roots due to long-term water stress is dependent on shoot organs. They showed a low capacity of the roots to synthesize ABA from carotenoids; however, plants were able to transport ABA basipetally to the roots. So why would ABA recirculate to the root? It is suggested that this process occurs to stimulate further ABA synthesis in the roots, perhaps acting as a higher level control mechanism at the plant scale.

### Conclusions

It is a well-known fact that the ABA concentrations are affected by drought and salt in plants. The present study proved that nutrient limitation or supply of an unfavourable N source (NH_4_^+^) besides salt application also resulted in changes of ABA pattern and transport in xylem as well as phloem.

Nutrient imbalance treatments had the largest effect on ABA in xylem, followed by that of leaf material. Treatment effects on variation in ABA concentrations were far less pronounced in phloem sap, axis tissue, and root tissue of *Ricinus* plants.

Aggregation of the data allowed the comparative analysis of ABA correlations with several physiological processes by means of regression analysis. The concentration of ABA in xylem sap correlated best with leaf stomatal conductance, (leaf) growth, and that in phloem sap, compared with other available parameters such as leaf ABA or ABA xylem flow.

A significant amount of ABA was transported in the phloem basipetally to the roots. On the basis of the present data, the large quantities of ABA transported towards the roots did not impede root growth; instead. root metabolism (synthesis/degradation) of ABA was positively correlated.

## Supplementary data

Supplementary data are available at *JXB* online.

Table S1 Summary of regression analysis for the effects on leaf stomatal conductance, ABA concentration in phloem sap, and ABA metabolism.

Table S2 Summary of regression analysis for the effects on different growth parameters.

Supplementary Data
