# Supplementary material for: ABA flow modelling in Ricinus communis exposed to salt stress and variable nutrition
Source: J Exp Bot. 2016 Jul 20;67(18):5301–11. doi: 10.1093/jxb/erw291 (PMC5049382; doi:10.1093/jxb/erw291)
Supplement: Supplementary Data [file supp_67_18_5301__index.html]

ABA flow modelling in Ricinus communis exposed to salt stress and variable nutrition — ABA flow modelling in Ricinus communis exposed to salt stress and variable nutrition — Supplementary Data 

# ABA flow modelling in *Ricinus communis* exposed to salt stress and variable nutrition

## Supplementary Data

Data files

- supplementary\_tables\_S1\_S2.pdf - Supplementary Data
