# Supplementary material for: ABA flow modelling in Ricinus communis exposed to salt stress and variable nutrition
Source: J Exp Bot. 2016 Jul 20;67(18):5301–11. doi: 10.1093/jxb/erw291 (PMC5049382; doi:10.1093/jxb/erw291)
Supplement: Supplementary Data [file supp_erw291_supplementary_tables_S1_S2.pdf]

Supplementary tables to:

ABA-FLOW MODELLING IN *RICINUS COMMUNIS* EXPOSED TO SALT STRESS AND VARIABLE NUTRITION

BY

ANDREAS D. PEUKE

Supplementary Table 1 Summary of regression analysis for the effects on A: leaf stomatal conductance (see Fig. 3) by ABA in leaves ($[ABA]_{lf}$), by ABA in xylem sap ($[ABA]_{xyl}$, root pressure), and by ABA flow in the xylem ($J_{X.ABA}$); on B: ABA concentration in phloem sap (see Fig. 5) by ABA in leaves ($[ABA]_{lf}$), by ABA in xylem sap ($[ABA]_{xyl}$, root pressure), and by ABA flow in the xylem from roots to leaves ($J_{X.ABA}$), calculated was additionally a model without intercept (/noint); and C: on ABA metabolism (synthesis or degradation) in roots (see Fig. 6) by ABA in phloem sap ($[ABA]_{phl}$), by ABA flow in phloem ($J_{P.ABA}$), and by ABA in roots ($[ABA]_{root}$)), calculated was additionally a model without intercept (/noint). Given are the estimates for intercept and slope ±SE, significance of estimates ($H_O = 0$) are indicated by asterisk: *: $p<0.05$; **: $p<0.01$; ***: $p<0.001$ (n.s.: not significant) and r^2; §: results shown as a regression curve in the figures.

Supplementary Table 2 Summary of regression analysis for the effects on different growth parameters (see Fig. 4). A: on leaf elongation rate by ABA in xylem sap ($[ABA]_{xyl}$, root pressure), by ABA in phloem sap ($[ABA]_{phl}$), and by ABA in leaves ($[ABA]_{lf}$); B: on leaf DW increment rate by ABA in xylem sap ($[ABA]_{xyl}$), by ABA in phloem sap ($[ABA]_{phl}$), and by ABA in leaves ($[ABA]_{lf}$); C: on shoot DW increment rate by ABA in xylem sap ($[ABA]_{xyl}$), by ABA in phloem sap ($[ABA]_{phl}$), and by ABA in leaves ($[ABA]_{lf}$); and D: on root DW increment rate by ABA in xylem sap ($[ABA]_{xyl}$), by ABA in phloem sap ($[ABA]_{phl}$) and by ABA in roots ($[ABA]_{rt}$).

Calculated were several models a full model and a model without intercept (/noint) as well as both with logarithmic models (y = ln(x)). Given are the estimates for intercept and slope ±SE, significance of estimates ($H_O = 0$) are indicated by asterisk: *: $p<0.05$; **: $p<0.01$; ***: $p<0.001$ (n.s.: not significant) and r^2; §: results shown as a regression curve in the figures.

Supplementary Table 1

	parameter	estimate	±SE	significance	r^2	
**A**	leaf stomatal conductance					
$[ABA]_{lf}$	intercept	624	111	**	0.31	
	slope	-296	179	n.s.		
$[ABA]_{xyl}$	intercept	581	48	***	0.70	§
	slope	-427	113	**		
$J_{X.ABA}$	intercept	571	70	***	0.44	
	slope	-0.06	0.03	n.s.		
**B**	ABA concentration in phloem sap					
$[ABA]_{lf}$	intercept	0.13	1.11	n.s.	0.47	
	slope	3.96	1.56	*		
$[ABA]_{xyl}$	intercept	0.82	0.52	n.s.	0.74	
	slope	4.83	1.01	**		
$J_{X.ABA}$	intercept	1.63	0.41	n.s.	0.06	
	slope	0.0002	0.0003	n.s.		
$[ABA]_{lf}$ /noint	slope	4.11	0.70	**	0.79	§
$[ABA]_{xyl}$ /noint	slope	5.97	0.76	***	0.87	§
$J_{X.ABA}$ /noint	slope	0.0011	0.0003	*	0.54	
**C**	ABA metabolism (synthesis / degradation) in roots					
$[ABA]_{phl}$	intercept	-416	466	n.s.	0.59	
	slope	529	148	**		
$J_{P.ABA}$	intercept	-890	755	n.s.	0.43	
	slope	3.16	1.20	*		
$[ABA]_{root}$	intercept	719	871	n.s.	0.005	
	slope	2824	13672	n.s.		
$[ABA]_{phl}$ /noint	slope	428	93	**	0.68	§
$J_{P.ABA}$ /noint	slope	1.90	0.56	**	0.53	§
$[ABA]_{root}$ /noint	slope	12388	7137	n.s.	0.23	

Supplementary Table 2

	parameter	estimate	±SE	significance	r^2	
**A**		leaf elongation rate				
$[ABA]_{xyl}$	intercept	0.90	0.23	**	0.30	
	slope	-0.87	0.54	n.s.		
$[ABA]_{phl}$	intercept	1.21	0.45	*	0.24	
	slope	-0.28	0.21	n.s.		
$[ABA]_{lf}$	intercept	1.12	0.29	n.s.	0.39	
	slope	-0.87	0.45	n.s.		
$\ln([ABA]_{xyl})$	intercept	0.17	0.28	n.s.	0.39	
	slope	-0.27	0.14	n.s.		
$\ln([ABA]_{phl})$	intercept	0.97	0.31	*	0.21	
	slope	-0.54	0.42	n.s.		
$\ln([ABA]_{lf})$	intercept	0.30	0.18	n.s.	0.55	
	slope	-0.39	0.14	*		
$[ABA]_{xyl}$/noint	slope	0.66	0.66	n.s.	0.35	
$[ABA]_{phl}$/noint	slope	0.24	0.11	n.s.	0.42	
$[ABA]_{lf}$/noint	slope	0.62	0.40	n.s.	0.25	
$\ln([ABA]_{xyl})$/noint	slope	-0.34	0.07	**	0.77	§
$\ln([ABA]_{phl})$/noint	slope	0.57	0.35	n.s.	0.28	
$\ln([ABA]_{lf})$ /noint	slope	-0.55	0.11	**	0.77	§
**B**		leaf DW increment rate				
$[ABA]_{xyl}$	intercept	1.31	0.29	**	0.49	
	slope	-1.44	0.65	n.s.		
$[ABA]_{phl}$	intercept	1.87	0.61	*	0.38	
	slope	-0.48	0.27	n.s.		
$[ABA]_{lf}$	intercept	1.40	0.48	*	0.24	
	slope	-0.91	0.72	n.s.		
$\ln([ABA]_{xyl})$	intercept	0.05	0.32	n.s.	0.65	
	slope	-0.46	0.15	*		
$\ln([ABA]_{phl})$	intercept	1.47	0.42	*	0.37	
	slope	-0.93	0.54	n.s.		
$\ln([ABA]_{lf})$	intercept	0.67	0.37	n.s.	0.12	
	slope	-0.23	0.29	n.s.		
$[ABA]_{xyl}$/noint	slope	0.64	0.97	n.s.	0.06	
$[ABA]_{phl}$/noint	slope	0.30	0.16	n.s.	0.37	
$[ABA]_{lf}$/noint	slope	0.87	0.56	n.s.	0.29	
$\ln([ABA]_{xyl})$/noint	slope	-0.48	0.07	**	0.88	§
$\ln([ABA]_{phl})$/noint	slope	0.67	0.51	n.s.	0.09	
$\ln([ABA]_{lf})$/noint	slope	-0.58	0.25	n.s.	0.48	
**C**		shoot DW increment rate				
$[ABA]_{xyl}$	intercept	1.38	0.33	**	0.44	
	slope	-1.47	0.74	n.s.		
$[ABA]_{phl}$	intercept	2.03	0.64	*	0.40	
	slope	-0.53	0.29	n.s.		
$[ABA]_{lf}$	intercept	1.50	0.52	*	0.24	

	slope	-0.99	0.78	n.s.		
$\ln([ABA]_{xyl})$	intercept	0.04	0.35	n.s.	0.65	
	slope	-0.49	0.16	*		
$\ln([ABA]_{phl})$	intercept	1.60	0.44	*	0.40	
	slope	-1.05	0.57	n.s.	0.40	
$\ln([ABA]_{lf})$	intercept	0.70	0.40	n.s.	0.12	
	slope	-0.26	0.31	n.s.		
$[ABA]_{xyl}$/noint	slope	0.72	1.05	n.s.	0.07	
$[ABA]_{phl}$/noint	slope	0.31	0.17	n.s.	0.36	
$[ABA]_{lf}$/noint	slope	0.92	0.60	n.s.	0.28	
$\ln([ABA]_{xyl})$/noint	slope	-0.51	0.08	***	0.87	§
$\ln([ABA]_{phl})$/noint	slope	0.70	0.55	n.s.	0.21	
$\ln([ABA]_{lf})$ /noint	slope	-0.63	0.26	n.s.	0.48	

**D**			root DW increment rate			
$[ABA]_{xyl}$	intercept	2.34	0.69	*	0.33	
	slope	-2.44	1.57	n.s.		
$[ABA]_{phl}$	intercept	3.37	1.37	n.s.	0.28	
	slope	-0.86	0.62	n.s.		
$[ABA]_{rt}$	intercept	1.95	0.95	n.s.	0.04	
	slope	-7.33	15.48	n.s.		
$\ln([ABA]_{xyl})$	intercept	0.23	0.87	n.s.	0.41	
	slope	-0.75	0.41	n.s.		
$\ln([ABA]_{phl})$	intercept	2.60	0.96	*	0.24	
	slope	-1.57	1.25	n.s.		
$\ln([ABA]_{rt})$	intercept	-0.43	2.56	n.s.	0.11	
	slope	-0.61	0.75	n.s.		
$[ABA]_{xyl}$/noint	slope	1.27	1.88	n.s.	0.07	
$[ABA]_{phl}$/noint	slope	0.55	0.31	n.s.	0.34	
$[ABA]_{rt}$/noint	slope	17.56	11.91	n.s.	0.27	
$\ln([ABA]_{xyl})$/noint	slope	-0.85	0.20	**	0.75	
$\ln([ABA]_{phl})$/noint	slope	1.26	0.99	n.s.	0.21	
$\ln([ABA]_{rt})$ /noint	slope	-0.49	0.15	*	0.63	
